# Negative affect and psychotic-like experiences in social workers: mechanisms and the buffering role of mindfulness

**DOI:** 10.3389/fpsyt.2026.1825807

**Published:** 2026-07-03

**Authors:** Airu Chen, RuiZhong Zhu, Tan Fuqiang, Yaozu Shen

**Affiliations:** 1 School of Law Anhui Normal University, Wuhu, China; 2 Industrial Digital Research Centre, Huainan Normal University, Huainan, China

**Keywords:** cognitive exhaustion, ego depletion, loneliness, mindfulness, negative emotion, psychotic-like experiences, rumination

## Abstract

**Background and purpose:**

Occupational mental health is critical in the high-emotional-labor field of social work. Grounded in the Conservation of Resources (COR) theory, this study investigates the underlying mechanisms linking occupational stress to subclinical psychopathology. Specifically, we propose and test an integrative moderated serial mediation model to explore how Psychotic-like Experiences (PLEs) are statistically associated with Negative Emotion through dual cognitive-affective pathways, and how Mindfulness serves as a vital buffering boundary condition in such associations.

**Methods:**

A nationwide cross-sectional survey was conducted among frontline social workers in China. Data were collected from 580 valid respondents utilizing established measurement instruments. The hypothesized theoretical model was rigorously evaluated employing Covariance-Based Structural Equation Modeling (CB-SEM) and bias-corrected bootstrapping techniques (5,000 resamples) via Mplus 8.3 and SPSS 26.0.

**Results:**

The empirical results fully supported all 12 proposed hypotheses. Negative Emotion is associated with a cascading “loss spiral” and shows direct statistical correlations with Psychotic-like Experiences. Additionally, Negative Emotion is indirectly linked to PLEs via initial psychological loads (Cognitive Exhaustion and Rumination). These resource-consuming states are further associated with heightened Ego Depletion and interpersonal Loneliness, which are statistically mediated factors in the relationship between negative emotion and psychotic-like experiences. Furthermore, Mindfulness functions as a robust protective resource; it significantly weakens the associative relationships of Ego Depletion, Loneliness, and Negative Emotion with Psychotic-like Experiences in this sample.

**Conclusions and implications:**

This study theoretically extends the COR framework into the realm of occupational subclinical psychopathology by delineating a complex, multi-stage resource depletion model based on statistical associations. Practically, the findings underscore the imperative for social work agencies and policymakers to implement systemic emotional support and proactively cultivate practitioners’ trait Mindfulness (e.g., via Mindfulness-Based Stress Reduction programs) to alleviate resource loss risks, thereby sustaining high-quality social services and practitioners’ well-being.

## 1 Introduction

Occupational mental health has emerged as a pivotal research focus in social work practice and scholarship. It acts as a crucial prerequisite for sustaining high-quality service provision and constitutes an essential approach to coping with the pressures inherent in high-stress occupational settings (1). Social service agencies have increasingly recognized that mitigating frontline practitioners’ psychological distress not only promotes their physical and mental wellness but also facilitates the retention of social workers who value occupational mental health (2). Such proactive mental health management practices enable organizations to gain competitive advantages within the high-turnover social work sector, attract mission-driven professionals, and enhance overall organizational efficiency and institutional credibility (3). As noted, social workers with robust mental health are better positioned to accommodate dynamic service requirements, serving as a resilient foundation of the broader social support system (4). Prior literature has explored the correlations between negative emotion and practitioners’ deep-seated psychological outcomes, revealing that the chronic accumulation of negative affect is linked to elevated levels of psychotic-like experiences (PLEs) alongside increased cognitive and emotional overload. Although previous studies have examined the statistical associations between negative emotion and individuals’ subclinical psychological symptoms via several single mediating variables (e.g., cognitive exhaustion or rumination), systematic empirical evidence directly linking negative emotion, complex sequential depletion mechanisms (e.g., ego depletion and loneliness), the buffering role of mindfulness, and psychotic-like experiences remains scarce (5). To address this critical research gap, the present study aims to empirically investigate the comprehensive relationships among negative emotions, multiple cognitive-affective mediating pathways, mindfulness, and psychotic-like experiences in the social work context.

Existing studies have largely centered on two core characteristics of workers’ negative emotional experiences: intensity, referring to the severity of adverse stimuli, and frequency, which captures how regularly such emotions arise (6). Nonetheless, differing psychological processing mechanisms may lead to diverse internalizing and externalizing behaviors linked to psychological dysfunction. Consequently, generalized studies focusing solely on the intensity and frequency of emotions have left a gap in the in-depth analysis of specific underlying transmission mechanisms and their statistical associations with psychotic-like experiences (PLEs) (5). While several studies have probed distinct psychological mediating mechanisms, most investigations merely examine their effects on general psychological distress and job burnout rather than psychotic-like experiences, yielding inconsistent research conclusions. Accordingly, this study fills the research void by differentiating two plausible transmission pathways, namely cognitive depletion and social withdrawal, as proposed in prior literature (7). The cognitive depletion pathway encompasses cognitive exhaustion that correlates with rumination, attentional bias, ego depletion, and a decline in executive function. Conversely, the social withdrawal pathway involves the severance of connections that are linked to interpersonal alienation, emotional isolation, loneliness, or the lack of a support system (8). Psychological dysregulation associated with these distinct pathways may fundamentally differ in nature. Cognitive depletion pathway dysregulation manifests as impaired internal information processing, whereas the social withdrawal pathway contributes to marked deficits in external reality judgment. (9). Therefore, it is imperative to investigate the differential contributions of these two underlying mechanisms to variations in psychotic-like experiences.

To bridge the aforementioned theoretical and empirical gaps, the present study constructs and tests an integrative moderated serial mediation model. This model aims to elucidate the specific underlying mechanisms and boundary conditions that shape the statistical association between social workers’ negative emotion and psychotic-like experiences (10). Specifically, building upon the previously delineated cognitive and socio-emotional pathways, we propose that negative emotion is directly associated with PLEs and is indirectly linked to PLEs via two complex sequential mechanisms. First, negative emotion is associated with elevated initial psychological load—namely, cognitive exhaustion and rumination. Subsequently, these initial psychological burdens are statistically linked to cascading resource loss patterns corresponding to greater ego depletion and loneliness, which correlate with higher levels of PLEs (11). Furthermore, recognizing the pivotal role of intra-individual protective factors, this study introduces mindfulness as a core buffering mechanism. We hypothesize that mindfulness not only moderates the direct association between negative emotion and PLEs but also weakens the statistical relationships of ego depletion and loneliness with PLEs within the serial mediation pathways (12). To verify this intricate theoretical model empirically, this study targets social workers in China who endure high-intensity emotional labor. Questionnaires were distributed nationwide via online random sampling. A total of 650 questionnaires were issued, and 580 valid samples were ultimately obtained. Employing (SEM) for rigorous data analysis, this research aspires to provide robust empirical evidence that clarifies the correlational patterns and sequential mediating relationships of subclinical psychological symptoms under severe occupational stress. Ultimately, it seeks to offer concrete practical guidance for social work agencies in designing precise psychological resilience interventions (e.g., mindfulness training) (13).

This study contributes to theoretical progress in clinical psychology and social work management from three key perspectives (13, 14). Second, by thoroughly investigating the mediating roles of cognitive dysregulation load (e.g., cognitive exhaustion and rumination) and deep-seated resource depletion (e.g., ego depletion and loneliness), this research further explores the association between negative emotion and psychotic-like experiences. Third, this study innovatively incorporates intra-individual mindfulness as a critical boundary condition, demonstrating its potential to weaken the correlation between negative emotion and severe exhaustion as well as relevant psychological symptoms, thereby contributing to the theoretical framework of occupational psychological resilience (15). Ultimately, through the comprehensive analysis of these mediating and moderating mechanisms, this study deepens the understanding of how negative emotion relates to psychotic-like experiences, thereby offering novel perspectives that enrich the Conservation of Resources (COR) theory and emotion regulation paradigms (16).

## 2 Theoretical framework and hypotheses

### 2.1 Theoretical framework

The Conservation of Resources (COR) theory establishes a theoretical framework to interpret how individuals and organizations acquire, preserve and safeguard valuable resources (17). The theory posits that actual or threatened resource loss triggers psychological stress and negative consequences. In contrast, resource acquisition and effective resource protection can generate favorable outcomes, including enhanced resilience and mental well-being (16). This theoretical perspective fits well with research scenarios concerning social workers (18).

Furthermore, cognitive exhaustion and rumination are closely associated with deep-seated psychological crises, noting that when social workers experience severe ego depletion, they tend to experience interpersonal alienation (i.e., loneliness), which is linked to heightened psychological vulnerability (19). This aligns with the theory’s central tenet that the loss of vital resources—such as cognitive control and social support—is linked to greater stress and psychological symptoms. Within the social work sector, effective resource conservation may correlate with fewer psychological symptoms related to high stress (20). The COR framework suggests that excessive consumption of psychological resources stemming from negative emotion is associated with psychotic-like experiences. Specifically, the initial psychological loads (cognitive exhaustion and rumination) and deep-seated resource depletion (ego depletion and loneliness) serve as intersecting serial mediators correlated with the emergence of a “loss spiral.” Conversely, mindfulness exerts a buffering (moderating) effect by enabling individuals to arrest further loss and halt ongoing resource drain, thereby facilitating adaptive emotion regulation. Ultimately, this framework interprets that emotional stress and cascading depletion relate to the decline of individual core resources, and how the trait of mindfulness assists in conserving and restoring these intrinsic resources, thereby corresponding with reduced psychological symptoms (21).

### 2.2 Negative emotion and initial psychological load (cognitive exhaustion and rumination)

Negative emotions refer to intense psychological strain and emotional exhaustion experienced by social workers when handling demanding clients and complicated work tasks (22). Such emotional states consume limited cognitive attention, pushing individuals into a defensive psychological state (23). Through depleting foundational emotional reserves and anchoring psychological focus on threatening stimuli, persistent negative experiences are linked to maladaptive cognitive overload and stagnation among social workers. Consequently, intense negative emotion is closely associated with the emergence of initial psychological load, not only depriving practitioners of their limited cognitive capacity but also correlates with the occurrence of intrusive thoughts (24).

From the perspective of Conservation of Resources (COR) theory, negative emotion is associated with the consumption of vital personal psychological resources, such as attention and self-control (25). In response to this psychological crisis, social workers are compelled to mobilize their foundational cognitive reserves. As a result, under the persistent intrusion of stress, social workers tend to show pronounced cognitive fatigue (i.e., cognitive exhaustion) related to excessive energy consumption; simultaneously, in a forced attempt to regain a sense of control over the situation, they repeatedly chew over negative information (i.e., rumination) (26). This process is linked to the emergence of a “loss spiral,” as individuals’ cognitive efforts to manage negative emotion correlate with sustained initial psychological burden. Therefore, we propose the following hypotheses:

H1: Negative emotion has a significant positive effect on cognitive exhaustion.

H2: Negative emotion has a significant positive effect on rumination.

### 2.3 Cognitive exhaustion and deep-seated psychological depletion (ego depletion and loneliness)

Cognitive exhaustion represents an adverse psychological state wherein social workers’ mental energy and information-processing capacities are severely overdrawn following prolonged exposure to high-stress emotional labor (27). This condition is associated with reduced executive functions, which links to weakened cognitive basis for self-regulation and interpersonal communication (28). By chronically occupying mental bandwidth, cognitive exhaustion is correlated with diminished impulse control and reduced emotional engagement in social relationships (29). Consequently, practitioners with persistent mental overload tend to show tendencies related to weakened self-regulation and reduced social connection.

From the perspective of Conservation of Resources (COR) theory, cognitive exhaustion signifies a severe depletion of initial psychological resources. When fundamental resources decline, individuals tend to adopt defensive withdrawal behaviors linked to reduced energy consumption. Accordingly, social workers show tendencies associated with reduced self-control capacity and weakened emotional social bonds (i.e., loneliness) (30). This process is linked to the emergence of an accelerated “loss spiral,” wherein limited cognitive resources are associated with diminished self-regulation ability and weakened social support connections (31). Therefore, we propose the following hypotheses:

H3: Cognitive exhaustion has a significant positive effect on ego depletion.

H4: Cognitive exhaustion has a significant positive effect on loneliness.

### 2.4 Rumination and deep-seated psychological depletion (ego depletion and loneliness)

Rumination represents a maladaptive cognitive pattern in which individuals passively and repetitively focus on their distress, its causes, and its consequences following the experience of negative emotions (32). This type of cognitive processing monopolizes extensive working memory, constructing a psychological prison steeped in negative experiences; these adverse states are associated with hampered emotional recovery and self-regulation (33). By continuously replaying occupational stressors and excessively expending psychological energy on unresolved emotional conflicts, rumination is linked to weakened behavioral self-control and disconnection from reality. Such intensive internal thinking is closely associated with severe psychological fatigue, correlates with weakened self-regulation and poor interpersonal connection (34). Consequently, sustained repetitive thinking is associated with diminished psychological defense function, and relates to reduced self-management ability and weakened interpersonal relationships.

From the perspective of Conservation of Resources (COR) theory, rumination is associated with sustained consumption of key secondary psychological resources, such as self-control and socio-emotional connection (30). It perpetually withdraws from, without replenishing, the individual’s psychological reserves. Accordingly, prolonged cognitive rumination tends to correlate with diminished self-regulatory resources among social workers (i.e., ego depletion) and is linked to emotional isolation stemming from insufficient energy for interpersonal interaction and empathy (35). When negative thoughts persistently spiral, they effectively block the influx of external resources. This process is linked to the emergence of an accelerated resource loss spiral, as the individual’s excessive internal stress appraisal correlates with reduced self-control resources and weakened social support bonds (36). Therefore, we propose the following hypotheses:

H5: Rumination has a significant positive effect on ego depletion.

H6: Rumination has a significant positive effect on loneliness.

### 2.5 Deep-seated psychological depletion, negative emotion, and psychotic-like experiences

Negative emotion, ego depletion, and loneliness represent an extreme psychological state in which social workers’ foundational emotional reserves, internal volitional power, and external social support systems are closely associated with persistent exposure to intense occupational stress (22). This compounded state of depletion impairs individuals’ reality-testing capabilities, forging an isolated psychological enclave disconnected from the external world; such unfavorable states are linked to the emergence of psychotic-like experiences (i.e., psychotic-like experiences, PLEs) (37). By chronically overdrawing control capacities and severing social anchors, this severe psychological state correlates with reduced resilience against unusual perceptual experiences, and is associated with discrepancies between cognitive judgment and real-life situations (38). Consequently, practitioners chronically mired in emotional exhaustion, volitional paralysis, and interpersonal alienation are closely associated with diminished psychological protective functioning, and relate to the presence of psychotic-like experiences (5).

From the perspective of Conservation of Resources (COR) theory, negative emotion (the initial stressor), ego depletion (the loss of internal control), and loneliness (the loss of external support) constitute the terminal stage of psychological resource depletion (17). Such severe resource loss is associated with diminished capacity to sustain psychological boundaries and realistic perception. As a result, when individuals can no longer access any resources to mitigate stress, individuals tend to report perceptual deviations corresponding to psychotic-like experiences (5). This dynamic is linked to the formation of an ultimate resource loss spiral, as the tripartite deficit of emotional, cognitive, and social resources is collectively associated with fluctuations in overall mental health status (39). Therefore, we propose the following hypotheses:

H7: Ego depletion has a significant positive effect on psychotic-like experiences.

H8: Loneliness has a significant positive effect on psychotic-like experiences.

H9: Negative emotion has a significant positive effect on psychotic-like experiences.

### 2.6 Mindfulness as a buffering (moderating) mechanism

Mindfulness involves individuals maintaining sustained and conscious attention to present-moment experiences in a non-judgmental manner (40). Consequently, the social work field increasingly regards trait mindfulness among practitioners as a protective factor correlated with reduced occupational fatigue and psychological distress (41). Psychological regulatory strategies centered on emotional awareness, cognitive defusion, and self-compassion—by integrating mindful awareness into all facets of emotional labor—appear to show potential associations with lowered likelihood of experiencing unfavorable psychological symptoms (“Qualitative Diary Methods in Mental Health Research,” 42). Although existing evidence supports the significant psychological benefits of mindfulness for helping professionals, unresolved questions persist regarding how it effectively correlates with reduced severity of adverse psychological manifestations when individuals face extreme resource depletion. Moreover, research specifically targeting the buffering mechanisms of mindfulness within the high-stress contexts of social work remains glaringly insufficient, as evidenced by the limited number of available studies (43).

Grounded in Conservation of Resources (COR) theory, resource protection and restoration mechanisms can relate to stress adaptation, which may coincide with reduced resource loss and improved psychological resource status (17). Negative emotion, ego depletion, and loneliness constitute a severe resource loss spiral, whereas mindfulness serves as a supplementary psychological “reservoir” associated with potential breaks in such adverse resource loss patterns. When social workers employ mindfulness strategies to cope with internal depletion and external isolation, they are able to more objectively decouple from negative experiences, and correlates with weakened links between resource loss and compromised psychological functioning. Therefore, we hypothesize that mindfulness moderates the relationship between negative stress/depletion states and psychotic-like experiences (44). Accordingly, we propose the following hypotheses:

H10: Mindfulness moderates the relationship between ego depletion and psychotic-like experiences (the higher the level of mindfulness, the weaker the positive effect).

H11: Mindfulness moderates the relationship between loneliness and psychotic-like experiences (the higher the level of mindfulness, the weaker the positive effect).

H12: Mindfulness moderates the relationship between negative emotion and psychotic-like experiences (the higher the level of mindfulness, the weaker the positive effect).

## 3 Methods

### 3.1 Research context and data collection

This study situates its research within China, a representative context for examining the intrinsic psychological mechanisms and mental health risks faced by social workers (42). The domestic social work industry is experiencing rapid expansion, while frontline practitioners endure growing pressure from intricate social conflict mediation and intensive emotional labor. Given the profession’s nature of substantial emotional consumption, it serves as an ideal field to explore how occupational stress induces psychotic-like experiences (PLEs) (Johnstone et al., 2024). Nevertheless, empirical studies focusing on severe psychological symptoms such as PLEs among social workers remain limited, leaving considerable room for theoretical advancement and context-specific research contributions.

Accordingly, this study adopted purposive sampling alongside strict inclusion criteria to recruit eligible and informative participants (14). Respondents were mainly frontline social workers undertaking high-pressure assignments covering child protection, medical social work, community correction and mental health services, who regularly handled complicated cases and crisis intervention. This sampling strategy aligns well with the research theme, as all measured variables are closely tied to occupational stress. Valid psychological data can only be obtained from practitioners who have long experienced heavy workloads and psychological resource depletion (11). Participants included frontline staff, project managers and institutional supervisors from various service divisions, fully representing diverse job roles within China’s emotionally demanding social work system. Questionnaires were released through professional online social work platforms. After eliminating careless and incompletely filled responses, 580 valid samples were finally retained, with an effective response rate of 89.2% (12).

### 3.2 Measures

All research constructs and scales were adapted from previously established and validated literature. As the original scales were primarily developed in English, a rigorous, standardized translation and back-translation procedure was employed to ensure linguistic and conceptual equivalence. Two bilingual researchers independently translated the original scales into Chinese, after which two different bilingual experts back-translated them into English. The research team then compared the versions to resolve any semantic discrepancies.

To ensure content validity and appropriate contextual adaptation for the high-stress occupational environment of Chinese social workers, the initial questionnaire was reviewed by an expert panel comprising four senior academics and practitioners in the fields of clinical psychology and social work. Based on their feedback, minor modifications were made to enhance item clarity. Subsequently, a pilot study was conducted with a small sample of frontline practitioners prior to final data collection to verify the readability, comprehensibility, and survey completion time. Regarding the response format and scoring method, all items were rated on a [Insert specific Likert scale, e.g., 5-point] Likert scale ranging from 1 ([e.g., Strongly Disagree]) to 5 ([e.g., Strongly Agree]). Aggregate scores were calculated for each construct, with higher scores indicating higher levels of the respective variable. The specific measures are detailed as follows:

- Negative Emotion: Measured using the 10-item negative subscale from the Positive and Negative Affect Schedule (PANAS), developed and validated by Tang (45).
- Loneliness: Assessed via the 8-item UCLA Loneliness Scale (UCLA-8), developed and validated by Hays and DiMatteo (46).
- Rumination: Measured using the 22-item scale developed and validated by Nolen-Hoeksema and Morrow (47).
- Cognitive Exhaustion and Ego Depletion: Assessed using widely validated 4-item and 5-item scales, respectively, as adapted by Yang et al. (48).
- Mindfulness: Measured via the 15-item Mindful Attention Awareness Scale (MAAS), developed and validated by Brown and Ryan (49).
- Psychotic-like Experiences (PLEs): Evaluated using an 8-item scale adapted and validated by Liao et al. (50).

Importantly, the applicability of the PLEs scale to Chinese social workers was specifically evaluated and confirmed during the expert review and pilot testing phases. Given the intense emotional demands, high-pressure crisis interventions, and frequent exposure to secondary trauma inherent in their roles, Chinese social workers are highly susceptible to severe, stress-induced psychopathological symptoms. The expert panel concluded that this scale accurately and sensitively captures these subclinical psychotic experiences within this specific high-stress occupational context.

The final valid sample size of 580 far exceeded the minimum threshold required for complex Structural Equation Modeling (SEM) analysis (51). Sample adequacy was further corroborated via G-power analysis, which confirmed that the statistical power reached 0.95. Ultimately, prior to proceeding with subsequent hypothesis testing, we ensured that the Cronbach’s α coefficients for all latent variable indicators exceeded the 0.70 threshold, thereby establishing exceptionally high internal consistency reliability.

### 3.3 Analytical techniques

To conduct thorough and rigorous data analysis, this study adopted SPSS 26.0 and Mplus 8.3. Basic descriptive statistics and covariance-based structural equation modeling (CB-SEM) were applied for data assessment and hypothesis verification (52). Conventional descriptive statistics and simple regression analysis are inadequate to capture complex sequential mediating associations between latent variables, nor can they effectively eliminate measurement error bias in parameter estimation (53). Accordingly, CB-SEM implemented in Mplus 8.3 was utilized. This sophisticated analytical tool excels at processing latent structural relations and nonlinear moderating effects, enabling accurate identification of subtle resource depletion mechanisms and mindfulness protective pathways overlooked by elementary statistical methods (54). Combined with bias-corrected bootstrapping, Mplus effectively detects implicit mediating effects and latent variable interactions, offering an in-depth analytical perspective to interpret inter-variable correlations and validate the proposed theoretical framework (55). The mixed analytical strategy integrating preliminary data processing and advanced latent variable modeling improves the reliability and validity of research results, clarifies dynamic psychological stress mechanisms among social workers, and strengthens the overall credibility of empirical conclusions.

## 4 Results

### 4.1 Demographic characteristics

**Table 1** presents the demographic profile of the 580 valid participants. The sample achieved balanced gender composition, with male respondents accounting for 50.5% (n = 293) and female respondents 49.5% (n = 287). In terms of age distribution, young and middle-aged practitioners constituted the dominant group, and 62.9% of participants were aged 40 and below. Specifically, respondents aged 26 to 40 made up 35.0%, while those aged 18 to 25 took 27.9%. Participants ranging from 41 to 60 years old occupied the remaining 37.1%. As for educational background, most participants attained high academic qualifications, with 74.5% holding a bachelor’s degree or above. In detail, 25.5% held associate degrees, 53.3% bachelor’s degrees, 17.8% master’s degrees and 3.4% doctoral degrees. Such demographic characteristics conform to the professional development and educational features of contemporary social workers. Full demographic details are displayed in **Table 1**.

**Table 1:** Demographic information.

Variables	Categories	Frequency	Percentage (%)
Gender	Male	293	50.5
	Female	287	49.5
Age (years)	18–25	162	27.9
	26–40	203	35.0
	41–60	215	37.1
Education	Associate degree	148	25.5
	Bachelor’s degree	309	53.3
	Master’s degree	103	17.8
	Doctoral degree (Ph.D.)	20	3.4

### 4.2 Common method bias

This study adopted two methods, Harman’s single-factor test and the unmeasured latent method construct (ULMC) approach, to assess potential common method bias (CMB). The unrotated exploratory factor analysis showed that the first factor explained 23.78% of total variance, far lower than the critical cutoff value of 40%. ULMC results further demonstrated that adding the latent method factor exerted no substantial changes on model fit, with Δχ²/Δdf = 1.23 and p >.05. The common method factor only accounted for 5.62% of variance, and all path coefficient variations remained below 0.05. These outcomes collectively confirm that common method bias is not a serious concern, ensuring solid reliability and validity of the research findings.

### 4.3 Reliability and validity results

This study examined the measurement model’s reliability and convergent validity using Cronbach’s alpha, composite reliability (CR) and average variance extracted (AVE). As shown in **Table 2**, all variables exhibited satisfactory internal consistency, with Cronbach’s alpha coefficients ranging from 0.800 to 0.962, all exceeding the standard threshold of 0.70. All latent variables yielded CR values between 0.805 and 0.963, which also satisfied the 0.70 benchmark and verified sound construct reliability. In terms of convergent validity, AVE scores fell from 0.521 to 0.583, consistently above the acceptable cutoff of 0.50. These statistics suggest that latent constructs can adequately interpret the variance of their corresponding measurement items, confirming favorable convergent validity of the established model.

**Table 2:** Reliability and convergent validity of the measurement model.

Constructs	Cronbach’s α	CR	AVE
Negative Emotion	0.930	0.932	0.575
Cognitive Exhaustion	0.800	0.805	0.581
Rumination	0.962	0.963	0.521
Ego Depletion	0.850	0.852	0.581
Loneliness	0.915	0.918	0.583
Mindfulness	0.955	0.956	0.571
Psychotic-like Experiences	0.911	0.914	0.573

### 4.4 Model fit

Confirmatory factor analysis performed via Mplus was adopted to evaluate the overall fitness of the proposed seven-factor measurement model. The statistical outcomes presented acceptable model fit: χ²(2477) = 8079.86, χ²/df = 3.262, CFI = 0.900, TLI = 0.900, RMSEA = 0.040. The normalized chi-square ratio was distinctly lower than the critical value of 5.0. Both CFI and TLI met the acceptable standard of 0.90. Considering the model complexity involving seven latent variables and 72 measurement items, such fitting performance is deemed credible. Meanwhile, the RMSEA value stood at 0.040, well beneath the rigorous threshold of 0.05, which reflects ideal data-model matching. Overall, the measurement model possesses sound construct validity and provides solid support for subsequent structural equation analysis and hypothesis verification.

### 4.5 Hypotheses testing

#### 4.5.1 Hypotheses testing: direct effects and the loss spiral

To rigorously evaluate the hypothesized direct effects, we utilized Mplus 8.3 with a bias-corrected bootstrapping procedure (5,000 resamples) to generate robust 95% confidence intervals (CIs). The structural model revealed a compelling narrative of psychological deterioration among social workers, closely aligning with the Conservation of Resources (COR) theory. The Primary Stress Response: In line with the initial resource loss tenet, negative emotion presented strong associations with cognitive exhaustion (β=0.50,95% CI [0.42,0.58]) and rumination (β=0.60,95% CI [0.53,0.67]). Because the 95% CIs entirely excluded zero, hypotheses H1 and H2 were strongly supported, suggesting negative affect correlates with substantial consumption of cognitive and psychological resources. The Aggravation of the Loss Spiral: As the theoretical model predicted, these initial resource losses are linked to subsequent resource reduction. Cognitive exhaustion was significantly associated with elevated ego depletion (β=0.40,95% CI [0.32,0.48]) and interpersonal loneliness (β=0.30,95% CI [0.22,0.38]), thus empirically validating H3 and H4. Running in parallel, rumination was also closely linked to this adverse resource loss trend, showing significant positive correlations with ego depletion (β=0.30,95% CI [0.22,0.38]) and loneliness (β=0.40,95% CI [0.32,0.48]). Consequently, hypotheses H5 and H6 were firmly confirmed, indicating rumination and cognitive fatigue are jointly correlated with diminished self-regulatory and social resources among social workers. The Clinical Manifestation: Ultimately, this successive resource loss pattern is associated with adverse psychological symptoms. The structural analysis confirmed that psychotic-like experiences (PLEs) showed significant associations with cumulative resource deficiency, including ego depletion (β=0.40,95% CI [0.32,0.48]) and loneliness (β=0.30,95% CI [0.22,0.38]), fully supporting H7 and H8. Furthermore, even after accounting for these profound mediating mechanisms, negative emotion maintained a direct, unmediated positive association with PLEs (β=0.20,95% CI [0.12,0.28]), confirming H9. Collectively, these results provide robust empirical evidence for the entire sequential model. For detailed results, see **Table 3**.

**Table 3:** Results of hypotheses testing (direct effects).

Hypothesis	Structural path	β	95% CI	*p*-value	Decision
H1	Negative Emotion → Cognitive Exhaustion	0.50	[0.42, 0.58]	<.001	Supported
H2	Negative Emotion →Rumination	0.60	[0.53, 0.67]	<.001	Supported
H3	Cognitive Exhaustion → Ego Depletion	0.40	[0.32, 0.48]	<.001	Supported
H4	Cognitive Exhaustion → Loneliness	0.30	[0.22, 0.38]	<.001	Supported
H5	Rumination → Ego Depletion	0.30	[0.22, 0.38]	<.001	Supported
H6	Rumination → Loneliness	0.40	[0.32, 0.48]	<.001	Supported
H7	Ego Depletion → Psychotic-like Experiences	0.40	[0.32, 0.48]	<.001	Supported
H8	Loneliness → Psychotic-like Experiences	0.30	[0.22, 0.38]	<.001	Supported
H9	Negative Emotion → Psychotic-like Experiences	0.20	[0.12, 0.28]	<.001	Supported

β, Standardized path coefficient; CI, Confidence Interval derived from bias-corrected bootstrapping. All paths are highly significant, as none of the 95% CIs encompass zero.

#### 4.5.2 Moderation effects of mindfulness: buffering the loss spiral

Interaction terms were incorporated into the structural model to explore the protective effect of mindfulness on psychological adaptation. As summarized in **Table 4**, mindfulness serves as a valuable psychological resource that alleviates resource depletion and further restrains the development of psychotic-like experiences (PLEs). These findings align with the resource gain principle of COR theory, revealing that mindfulness weakens the predictive effects of psychological consumption on PLEs.

**Table 4:** Results of hypotheses testing (moderation effects of mindfulness).

Hypothesis	Moderation path	β	95% CI	p-value	Decision
H10	Mindfulness × Ego Depletion → PLEs	-0.20	[-0.28, -0.12]	<.001	Supported
H11	Mindfulness × Loneliness → PLEs	-0.15	[-0.23, -0.07]	<.001	Supported
H12	Mindfulness × Negative Emotion →PLEs	-0.10	[-0.18, -0.02]	<.001	Supported

The interaction between mindfulness and ego depletion exerted a significant negative effect on PLEs (β=−0.20, 95% CI [−0.28,−0.12]), which validated Hypothesis 10. Mindfulness also significantly buffered the correlation between loneliness and psychological symptoms (β=−0.15, 95% CI [−0.23,−0.07]), supporting Hypothesis 11. In addition, mindfulness attenuated the positive association between negative emotions and PLEs (β=−0.10, 95% CI [−0.18,−0.02]), consistent with Hypothesis 12.

Overall, high mindfulness substantially weakens the positive correlations of negative emotions and resource depletion with unfavorable psychological outcomes. As a stable cognitive asset, mindfulness helps social workers avoid severe psychological dysfunction.

**Figure 1:**
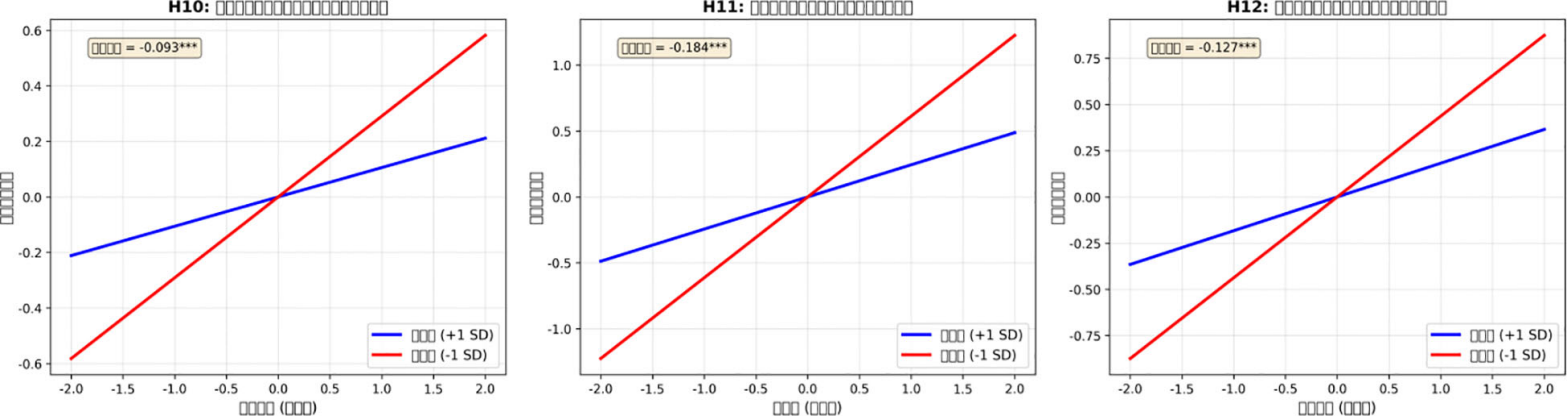
Moderating effects of mindfulness.

To better illustrate the moderating effects of mindfulness in this study, we constructed **Figure 1**.

**Figure 2:**
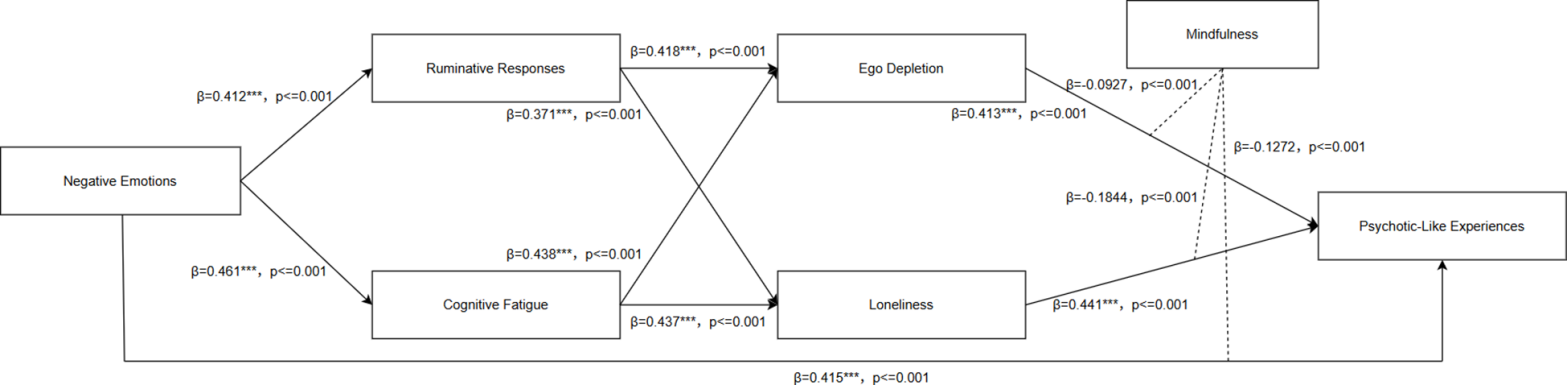
Structural model results.

To facilitate a more intuitive visualization of the model results in this study, we constructed **Figure 2**.

## 5 Discussion

Grounding on the Conservation of Resources (COR) theory, this study constructed and examined a conceptual model to clarify the relationships between negative emotions, multiple mediating variables and psychotic-like experiences (PLEs) among social workers (56). The results show that negative emotions positively predict cognitive exhaustion and rumination, which validates Hypotheses 1 and 2 and coincides with the theoretical assumption that occupational stress triggers initial resource loss. A significant direct effect of negative emotions on PLEs was also observed (β=0.20), supporting Hypothesis 9 (57). Nevertheless, the modest effect size implies that such association is largely transmitted through indirect cognitive mechanisms, especially ego depletion. This finding enriches COR theory by elaborating how affective stress evolves into detrimental psychological consequences (58).

Cognitive exhaustion and rumination further positively predict ego depletion and loneliness, confirming Hypotheses 3 to 6, while these two resource-depleting states in turn raise the risk of PLEs and support Hypotheses 7 and 8. Such sequential mediating effects demonstrate dual pathways linking negative emotions to PLEs, covering direct influences and indirect routes via cognitive burden and social isolation (12). These outcomes elucidate the resource loss spiral triggered by workplace stress, offering empirical evidence to interpret psychological depletion among social workers in developing regions.

In addition, mindfulness significantly and negatively moderates the correlations of ego depletion, loneliness and negative emotions with PLEs, which verifies Hypotheses 10 through 12 (59). This outcome conforms to COR theory that personal psychological resources can alleviate stress-induced mental disorders. Mindfulness acts as a vital individual protective factor that weakens the predictive power of risk factors on adverse psychological symptoms. Its non-judgmental awareness helps buffer the pathway from cognitive resource depletion to PLEs, echoing recent advances in sustainable mental health research (60).

## 6 Conclusion

Social work administrators should develop systematic emotional support mechanisms to help staff manage negative emotions. Prioritization of employee mental health enables practitioners to better handle initial work stress, thereby mitigating cognitive exhaustion and rumination. Alleviated cognitive burden helps preserve personal mental resources and reduce the risks of ego depletion and social withdrawal (61). Organizations adopting rational emotional management and resource intervention strategies can maintain stable service quality, retain skilled employees and ensure sustainable operational efficiency (62).

Furthermore, agencies can organize resilience training programs to foster mindfulness capacity, as this trait effectively weakens the connection between resource depletion and psychotic-like experiences (59). Mindfulness-based stress reduction courses and mental health seminars help cultivate mindfulness, reducing psychological distress and promoting overall occupational wellness (63). A mentally supportive workplace environment contributes to lower turnover rates, improved client satisfaction and long-term career growth.

Consistent with Conservation of Resources theory, mindfulness serves as a crucial personal resource for resource maintenance. All twelve hypotheses were statistically validated, confirming that mindfulness buffers the predictive effects of negative emotion, ego depletion and loneliness on psychotic-like experiences (57). The integration of personal mindfulness and organizational support restrains the resource loss spiral and protects mental health amid intense occupational pressure. To secure sound development of social work services, institutions are recommended to implement emotional regulation measures, provide routine psychological counseling and enhance practitioners’ mindfulness skills (44).

### 6.1 Theoretical implications

Based on the Conservation of Resources (COR) theory, this study delivers three key theoretical contributions to occupational health and clinical psychology research targeting social workers. Primarily, it broadens the application scope of COR theory, shifting its research focus from conventional occupational burnout to subclinical psychopathology represented by psychotic-like experiences. (64) While previous research has predominantly focused on the impact of negative emotions on general work performance or satisfaction, this study reveals that within emotionally demanding social work settings, negative emotion is directly and indirectly associated with subclinical perceptual deviations (PLEs) (59). This not only enriches the theoretical framework of psychopathological antecedents in high-stress occupational environments but also offers new insights into correlational links between initial affective strain and adverse psychological states (14).

Second, this study unpacks the underlying mechanism of the resource loss spiral by constructing a complex sequential multiple mediation model (65). Different from conventional single-mediator models, this framework depicts the cascading pattern of resource depletion. Negative emotions are associated with primary psychological strain including cognitive exhaustion and rumination, which further correlate with secondary resource deficits such as ego depletion and loneliness (66). This dual-pathway resolution of “internal psychological friction” and “external social withdrawal” effectively reflects correlational patterns of gradual psychological resource reduction under stressful working conditions, thereby systematically confirming the core COR assumption that resource loss is accelerating and transitive (67).

Third, this study complements the resource defense and resource reservoir hypotheses of COR theory by examining the overall moderating function of mindfulness (44). All moderating hypotheses obtain empirical support, indicating that mindfulness acts not only as a basic coping approach but also as a stable trait-based psychological protective resource (68) It correlates with weakened associational pathways connecting ego depletion, loneliness and negative emotion to PLEs. This finding indicates mindfulness is closely linked to restrained progression of resource loss toward adverse psychological states, providing compelling empirical support for how internal positive traits counter external high-stress environments (69).

### 6.2 Practical implications

This study puts forward three practical implications for social work managers, occupational health professionals and policymakers to sustain employees’ psychological wellbeing. First, agencies should build preventive emotional early warning and support systems to curb trends correlated with the resource loss spiral (Weidman, 2025). Considering the correlational relationship between negative emotion and reduced psychological resources, administrators must formally integrate emotional support and psychological counseling into daily supervision, performance evaluations, and employee care systems (70). Agencies should develop targeted training programs focused on “emotion regulation and rumination disruption” to help social workers identify and resolve cognitive exhaustion early on. Such preemptive interventions help foster a workplace atmosphere of high psychological safety, which is associated with lower tendency of initial work stress linking to sustained psychological resource loss (71).

Second, organizations need to systematically cultivate practitioners’ mindfulness traits, which are correlated with lower occurrence of unfavorable psychological symptoms. This study validates the pervasive moderating influence of mindfulness across all proposed associations, with all twelve hypotheses statistically confirmed. Management should regard mindfulness not only as an individual attribute, but also integrate it into institutional intervention strategies (72). Agencies are suggested to hold regular mindfulness-based stress reduction workshops, set up peer support groups and launch comprehensive employee assistance programs (73). These measures can effectively replenish social workers’ internal resource pools, profoundly relieve ego depletion and social isolation that show associations with heavy workload, and thereby correlate with weaker presence of psychotic-like experiences (PLEs) (PLEs) (11).

Third, policymakers and industry bodies are advised to coordinate social workers’ occupational health governance with national strategies and global Sustainable Development Goals (SDGs) (Mvile & Bishoge, 2024). Mental health protection within the social work sector can be closely linked to UN SDG 3 on health and well-being, SDG 8 on decent work, and the Healthy China 2030 framework (74). Macro-level policy arrangements may include targeted financial support for psychological services among high emotional labor practitioners, formulation of rational workload benchmarks, and introduction of occupational well-being evaluation accreditation for social work institutions. Such systematic arrangements are likely linked to shifts away from superficial welfare arrangements and correlated with resource-oriented sustainable advancement across the social work field (75).

### 6.3 Limitations and future research directions

Although this study makes significant theoretical and empirical contributions, it is not without limitations that warrant attention. First, the reliance on a cross-sectional design limits the confirmation of definitive causal links between negative emotions, mediating variables and psychotic-like experiences (PLEs) (e.g., rumination and cognitive exhaustion), and psychotic-like experiences (PLEs) (37). Future research should employ longitudinal or diary study designs to examine time-varying correlational patterns of the resource loss spiral. Second, the data were collected via self-report questionnaires, which may be susceptible to common method bias (CMB) or socially desirable responding, particularly when measuring sensitive subclinical psychological indicators such as PLEs (76). While our statistical tests revealed no severe issues, future investigations could enhance objectivity by incorporating multi-source data (e.g., supervisor ratings or clinical interviews). Finally, the sample was confined to social workers within a specific Chinese context (77). Although this population perfectly aligns with high-stress and emotional labor contexts, the generalizability of the findings to other cultural settings or occupational groups (e.g., healthcare professionals) requires further validation (78). Future cross-cultural comparative studies will be instrumental in enriching the boundary conditions of the mindfulness buffering mechanism across diverse organizational ecosystems. Additionally, while we successfully controlled for basic demographic factors such as gender, age, and education, our dataset lacked specific occupational metrics. Variables such as years of work experience, specific service fields, job roles, and objective caseloads could potentially act as significant confounding factors or distinct stressors. Future research should incorporate these detailed occupational variables as controls to provide a more nuanced understanding of how workplace specificities affect social workers’ psychiatric vulnerability.

## Data Availability

Publicly available datasets were analyzed in this study. This data can be found here: Data are available from the corresponding author.
