# Supplementary material for: Negative affect and psychotic-like experiences in social workers: mechanisms and the buffering role of mindfulness
Source: Front Psychiatry. 2026 Jul 3;17:1825807. doi: 10.3389/fpsyt.2026.1825807 (PMC13375970; doi:10.3389/fpsyt.2026.1825807)
Supplement: Supplementary file 1 [file SupplementaryFile1.docx]

Kegelaers, J., Trotter, M. G., Watson, M., Pedraza-Ramirez, I., Bonilla, I., Wylleman, P., Mairesse, O., & Van Heel, M. (2024). Promoting mental health in esports. *Frontiers in Psychology*, *15*(15), 1342220. <https://doi.org/10.3389/fpsyg.2024.1342220>

Thompson, R. N. (2015). SOCIAL SERVICE. *American Journal of Orthopsychiatry*, *11*(2), 378–380. <https://doi.org/>10.1111/j.1939-0025.1941.tb05816.x

McNamara, R., Nour, N., Mannix, M., Browne, J., & Donohue, S. (2024). Population Health Management effectiveness in the primary healthcare setting: A systematic review. European Journal of Public Health, 34(Suppl. 3), ckae144.1154. https://doi:10.1093/eurpub/ckae144.1154

Hill, N. G., & Forney, K. J. (2025). Negative emotion differentiation, but not gastric interoception, is linked to "feeling fat" among women with elevated eating pathology. *Eating Behaviors*, *57*. <https://doi.org/>10.1016/j.eatbeh.2025.101964

Cedrún-, E., Núez-Ríos, J. E., Sánchez-García, J. Y., Sosa-Gómez, G., & Rojas, O. (2025). Structural Equation Modeling for Analyzing Pro-Environmental Behavior in Switzerland. *Sustainability (2071-1050)*, *17*(8). <https://doi.org/>10.3390/su17083624

Guillén, V. (2024). Psychological interventions for family members of people with psychological disorders with emotional dysregulation: Introduction to special section. *Family Process*, *63*(4). <https://doi.org/>10.1111/famp.13090

Denninger, J. W., Joss, D., Romero, P. M., Khalsa, S. B. S., Hoge, E. A., Bhasin, M., Lazar, S. W., Dusek, J. A., Macklin, E., Libermann, T., Fricchione, G. L., & Benson, H. (2025). Psychological assessments, allostatic load and gene expression analyses in a randomized controlled trial comparing meditation, yoga, and stress education. Frontiers in Psychology, 16, 1653242. https://doi.org/10.3389/fpsyg.2025.1653242

Saija, E., Pallini, S., Baiocco, R., & Ioverno, S. (2025). Children's Narratives of Sad Events: Attachment Security and Psychopathological Symptoms. *Psicothema*, *37*(2). <https://doi.org/>10.70478/psicothema.2025.37.16

Liu, J., Siano, P., & Wu, K. (2025). Exploration of drivers for energy regulatory process reengineering in the digital era: The grounded theory approach. *Energy*, *316*(000). <https://doi.org/>10.1016/j.energy.2025.134620

Elster, A., & Sagiv, L. (2025). Personal Values and Cognitive Biases. *Journal of Personality*, *93*(6). <https://doi.org/10.1111/jopy.13001>

Shtulman, A., & Young, A. G. (2023). The development of cognitive reflection. *Child Development Perspectives*, *17*(1), 48–54. <https://doi.org/10.1111/cdep.12476>

Song, K., Xiao, Y., He, L., & Du, P. (2025). From proactive procrastination to proactive innovative behavior: the psychological transformation pathway based on conservation of resources theory. *Current Psychology*, *44*(1), 602–615. <https://doi.org/10.1007/s12144-024-07177-w>

Liu, B., Shah, T. A., & Shoaib, M. (2024). Creative leadership, creative mindset and creativity: a self-regulatory focus perspective. *Current Psychology*, *43*(29). <https://doi.org/>10.1007/s12144-024-06066-6

Brien, C. S., & Hutchins, T. L. (2024). The Key to Successful Social Interactions. *Topics in Language Disorders*, *44*(4), 20. <https://doi.org/10.1097/TLD.0000000000000353>

Talia, A., Kling, L., & Taubner, S. (2024). bersichtsarbeiten / Review Articles. Die Entwicklung von epistemischerWachsamkeit und epistemischem Vertrauen über die Lebensspanne: Perspektive aus der empirischen Forschung zum selbstregulierenden sozialen Lernen / The Development of Epistemic Vigilance and Epistemic Trust Across the Lifespan: Perspectives from Empirical Research on Self-Regulatory Social Learning. *Praxis der Kinderpsychologie und Kinderpsychiatrie*, *73*(4). <https://doi.org/>10.13109/prkk.2024.73.4.362

Cobb-Clark, D. A., Dahmann, S. C., Kamhfer, D. A., & Schildberg-Hrisch, H. (2025). Schooling and Self-Control. *Journal of Economic Behavior & Organization*, *237*(000). <https://doi.org/10.1016/j.jebo.2025.107147>

Hwang, W. C., & Kong, Y. (2024). Addressing Systemic Racism in Mental Health Care. *Review of General Psychology*, *28*(4), 315–325. <https://doi.org/10.1177/10892680241289349>

Cheng, S., Liu, S., Zhang, X., Zhou, J., & Feng, X. (2025). Mindfulness and media-driven prosociality: effects of trait and state mindfulness on responses to conflict photojournalism. Frontiers in Psychology, 16, Article 1619688. <https://doi.org/10.3389/fpsyg.2025.1619688>

Yang, H., Zeng, Y., Xing, H., & Hu, P. (2026). Fatigued by uncertainties: Exploring the cognitive and emotional costs of generative AI usage. *International Journal of Information Management*, *87*, 103010. [https://doi.org/10.1016/j.ijinfomgt.2025.103010](https://doi.org/https://doi.org/10.1016/j.ijinfomgt.2025.103010)

Ansah, E. W., Rodriguez, D., & Burnette, C. B. (2024). Editorial: The use of Structural Equation Modeling (SEM) methods in eating behavior research. *Frontiers in Psychology*, *15*(000), 2. <https://doi.org/10.3389/fpsyg.2024.1378515>

Bailon, L. L., Anadon, C., Chaurio, R., Mine, J., Plappert, N., Antonia, S., & Conejo-Garcia, J. (2024). Novel tumor-specific Vd1/3 gd CAR T cells effectively control solid tumors. The Journal of Immunology, 21(1_Supplement), 0047_4696.<https://doi.org/10.4049/jimmunol.212.supp.0047.4696>

Langenbucher, A., Wendelstein, J., Cayless, A., Olsen, T., Hoffmann, P., & Szentmáry, N. (2025). Jackknife and bootstrapping resampling techniques to evaluate the precision of lens formula constants. *Acta Ophthalmologica (1755375X)*, *103*(6). <https://doi.org/10.1111/aos.17522>

Fryland, L. R., Tokle, R., Andreas, J. B., & Brunborg, G. S. (2024). Sexting and Mental Health in Adolescence: A Longitudinal Study. *Journal of Adolescent Health*, *75*(4), 7. <https://doi.org/>10.1016/j.jadohealth.2024.04.018

Qi, M., Shahwar, Y., Wang, Y., Wang, H., Jin, Y., Bosch-Bayard, J. F., Morales, C. L., Valdes-Sosa, P. A., & Bringas-Vega, M. L. (2025). NeuroEPO in the Neuroprotection of Parkinson's Disease: A Study on EEG Spectrum Regulation and Mediation Mechanism. *International Journal of Psychophysiology*, *213*. <https://doi.org/10.1016/j.ijpsycho.2025.112664>

Kugler, K. G., & Coleman, P. T. (2024). One style does not fit all: the relationship of mediator behavioral adaptivity to mediator empowerment, efficacy, and satisfaction. *International Journal of Conflict Management*, *36*(1), 166–190. <https://doi.org/10.1108/IJCMA-03-2024-0060>

Mingjie, G., Jun, M., & Huirong, T. (2025). The double-edged sword effect of generative artificial intelligence usage on employee creativity: a perspective of conservation of resources theory. *Current Psychology*, *44*(10). <https://doi.org/>10.1007/s12144-025-07806-y

Ulrich, R., Irmgard, D. L. V., Eikmeier, V., Günther, F., & Kaup, B. (2024). Mental association of time and valence. *Memory & Cognition*, *52*(2). <https://doi.org/10.3758/s13421-023-01473-9>

Lam, J. A., Seo, V., Overhage, L. N., Keane, E. P., Dobbins, A. R., Granoff, M. D., Progovac, A. M., & Amonoo, H. L. (2026). Positive psychological well-being and psychological distress in higher education students. *Journal of affective disorders*, *394*(pa), 120550. <https://doi.org/10.1016/j.jad.2025.120550>

Suszek, H., Kopera, M., & Jakubczyk, A. (2025). The multiple self and psychological openness. *Frontiers in Psychology*, *15*, 1441953. <https://doi.org/10.3389/fpsyg.2024.1441953>

Glazzard, J., & Bostwick, R. (2025). What is Mental Health? *A Whole School Approach to Mental Health and Well-being*, 3–23. <https://doi.org/10.4324/9781041056485-2>

Miao, A. S. (2024). Artworks and psychological reparation. *Psychotherapy Section Review*(70). <https://doi.org/10.53841/bpspsr.2024.1.70.32>

Shen, D., Schaan, E., & Ferraro, S. (2024). CMB lensing power spectrum without noise bias. *Physical review,D*(4 Pt.C), 110. <https://doi.org/>10.1103/PhysRevD.110.043523

Zhou, Y., & Hommel, B. (2024). On the Generalizability of the Bodily State Effect on Creativity. *Creativity Research Journal*, *36*(4), 705–720. <https://doi.org/10.1080/10400419.2023.2171339>
